# Why and How Are Infants with Hutchinson–Gilford Progeria Syndrome Born Without Severe Manifestations?

**DOI:** 10.3390/medsci13040321

**Published:** 2025-12-15

**Authors:** Mariia A. Erokhina, Ekaterina A. Vorotelyak, Andrey V. Vasiliev, Vepa K. Abdyev

**Affiliations:** 1 Institute of Gene Biology, Russian Academy of Sciences, Vavilov Str. 34/5, 119334 Moscow, Russia; maryckx1994@mail.ru; 2 Laboratory of Cell Biology, N.K. Koltzov Institute of Developmental Biology, Russian Academy of Sciences, Vavilov Str. 26, 119334 Moscow, Russia; vorotelyak@yandex.ru (E.A.V.);

**Keywords:** Hutchinson-Gilford progeria syndrome (HGPS), Lamin A, progerin, Lamin B1, embryonic development, epithelial-to-mesenchymal transition (EMT), mechanotransduction

## Abstract

Children with Hutchinson–Gilford progeria syndrome (HGPS) are born without height and weight abnormalities, and postnatal development is delayed from two months of age. The pathophysiological manifestations of HGPS can be categorized into the three tissue systems that are primarily affected: bone and cartilage, the smooth muscular layer of the vasculature, and the dermis layer. To understand the biology of the syndrome’s complications resulting from the inherited dominant mutation of the *LMNA* gene, HGPS has to be considered in embryogenesis. Since the development of the primarily affected HGPS tissues involves a simultaneous contribution of mesodermal and neural crest cells, we hypothesized that the stochastic and heterogeneous coexistence of mesoderm and neural crest cells might be crucial for the onset and manifestation of HGPS. In addition, the expression of Lamin A and/or progerin during embryonic development tends to accumulate in the cell nucleus, causing the syndrome manifestation. Then, how and why are infants with the *LMNA* gene mutation born without severe deviations? Migration is a distinguishing property of mesoderm and neural crest cells, so that they are continuously subjected to mechanical stimuli throughout development and require normal lamina function. However, the viscoelastic property and the mechanosensor capability to respond to mechanical stress of the HGPS cell nucleus are disturbed. Despite the presence of progerin in development, we assume that high levels of Lamin B1 in cells determine the delayed onset of HGPS after birth. We also hypothesized that progerin toxicity could be managed and prevented, potentially allowing for rescue by the presence of Lamin B1.

## 1. Introduction

Children with Hutchinson–Gilford progeria syndrome are usually born with no abnormalities in height or weight [1,2,3]. However, a slight delay may be observed from the age of two months [1]. This physical retardation then progresses rapidly from the age of one year onwards, accompanied by other symptoms [1,2,4]. The time of manifestation and the severity of symptoms may vary slightly from case to case [4]. Apart from growth delay, patients develop signs of premature aging, affecting only some tissues, involving bones, skin, and derivative alterations, along with cardiovascular diseases, whereas cognitive development and a range of other tissues and organs stay unaffected [2,3,4,5,6,7].

The disease is caused by a heterozygous de novo point mutation within the 11th exon of the *LMNA* gene on chromosome 1 [8,9]. Children with Hutchinson–Gilford progeria syndrome are born to unaffected parents, as their parents’ DNA sequencing suggests a sporadic emergence of the mutation in the parents’ germline, with a statistical prevalence of paternal origin [10,11]. Although the HGPS mutation does not change the amino acid sequence, it results in the activation of a cryptic splice site [8,9]. Apart from the most common heterozygous C1824 to T1824 silent substitution, other variants of nucleotide transition adjacent to the classic nucleotide position of HGPS mutation were observed, causing the same alternative splice site activation and HGPS phenotypes of varying severity [9,12]. The *LMNA* gene encodes different forms of lamins A and C, which are components of the nuclear lamina protein complex underlying the nuclear envelope [13]. Mature Lamin A is a result of a range of post-translational modifications of prelamin A [13], which include steps of farnesylation and the subsequent cleavage of the C-terminus by Zmpste24 endoprotease [9,14,15]. This last process is disrupted in the case of a mutant form of prelamin A, as the alternatively spliced RNA lacks 150 nucleotides; thus, the translated protein is depleted of a 50-amino acid section, which is the target site of Zmpste24 endoprotease [9,14]. Therefore, mutant Lamin A, or progerin, represents the carboxymethylated and farnesylated shortened (614aa) form of normal Lamin A (646aa) [14]. The carboxymethylated and farnesylated C-terminus of progerin permanently incorporates into the inner nuclear membrane, altering the nuclear membrane structure. Consequently, patient-derived cells in culture exhibit nuclear malformation and rupture, leading to DNA damage [16] and cell senescence [17].

**Figure 1:**
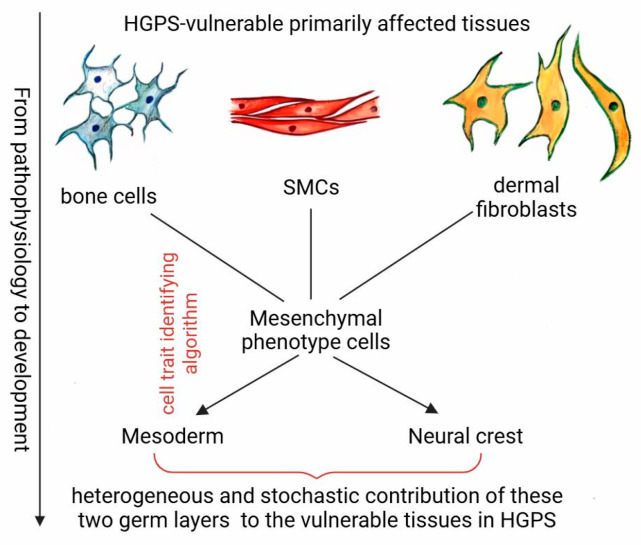
Schematic illustration of tracing back to the germinal layers from HGPS pathophysiology.

The pathophysiological manifestation of HGPS indicates that complications resulting from the inherited autosomal dominant mutation of the *LMNA* gene can be categorized into the three tissue systems that are primarily affected: bone and cartilage, the smooth muscular layer of the vasculature, and the dermis layer. If the development of these three tissue systems is addressed to embryogenesis, the origin of these tissues belongs to the mesoderm germ layer and the neural crest concurrently (Figure 1). Consequently, the affected tissues in HGPS patients can be considered as a cell population of heterogeneous origin from these two germ layers, and a stochastic contribution to the developing tissues. Therefore, the stochastic and heterogeneous origin of tissue development in the embryogenesis of bone and cartilage, vascular smooth muscles, and the dermis layer from mesoderm and neural crest cells might be crucial for the onset and the manifestation of the HGPS.

## 2. Embryonic Development in Relation to HGPS Manifestation

To support the proposed hypothesis regarding the embryonic progenitors of affected tissues in HGPS patients and the possible moment of syndrome onset during development, early embryonic development is considered. Since a de novo point mutation within the 11th exon of the *LMNA* gene, causing a cryptic splice site activation [8,9], results in the accumulation of nuclear lamina progerin protein, the HGPS phenotype of different severity manifests due to the progerin toxicity [9,12]. Furthermore, the specific expression patterns of the heterozygous *LMNA* gene in affected tissues most likely determine their vulnerability to HGPS. In addition, since the *LMNA* gene activation connotes cell differentiation, tracing the route back to the development of progenitor stem cells from the pathophysiology of HGPS points to the possible onset of progerin accumulation and its behavior during development. Accordingly, to delineate vulnerable cell types in embryogenesis, we have developed an algorithm for identifying cell trait-bearing mesenchymal phenotype cells that are subjected to continuous mechanical stress on the nuclear lamina (Figure 1).

### 2.1. Lamin A Contributes to Trophectoderm and Hypoblast Specification in Preimplantation Development

Before the formation of the three germ layers, human embryonic development proceeds through blastocyst formation, initiated by the segregation of outer trophectoderm (TE) cells and the inner cell mass (ICM) [18]. Presumptive TE cells initiate E-cadherin-mediated cell–cell adhesion to compact them, so that the PAR3-PAR6-aPKC polarization and apical cortex of polarized F-actin determine TE specification [19]. As a result of the cell polarization and F-actin contractility, the presumptive TE cells develop tension and mechanical forces, which are further transmitted to the nucleus, so that the wrinkled nuclear morphology at the mouse 2-cell stage embryo develops into a Lamin A/C-based spherical nucleus [20,21]. In other words, mouse and human embryo compaction and contractility of presumptive TE cells drive the enrichment of Lamin A/C at nuclear lamina, consistent with low cytoplasmic active Yap and the highest levels of Cdx2 [20], which specify TE formation. Furthermore, the differentiation of induced pluripotent stem cells (IPSCs) derived from HGPS patients into trophoblast stem cells revealed the inability of these cells to activate the TE gene markers *GATA3* and *KRT7* (unpublished data, as disclosed by the authors). TE differentiates into syncytiotrophoblasts (STBs) during embryo implantation [22]. The formation of human STBs is specified by nuclear enlargement and syncytialization, which are facilitated by the contribution of Lamin A [22]. Disruption of the *LMNA* gene in STBs results in a reduction in the percentage of multinucleated cells [22], indicating an interruption of placental development. In addition, the implantation is an active process through invasion of the endometrium by mural trophoblasts, which upregulate the cell migration (*LCP1*, *EFHD2*, and *FMNL2*) genes [19]. As will be discussed further, the cell migration initiates a sequence of mechanical cues that affect the cell nucleus through nuclear lamina, especially Lamin A. Taken together, the *LMNA* gene is activated in the first cell segregation in a preimplantation embryo [20], so that progerin toxicity might intervene TE specification and/or STB nuclear syncytialization, which might indicate the incapability of the HGPS embryo to implant and/or abruptly stop the placental development. Such an observation suggests that the de novo point mutation within the 11th exon of the *LMNA* gene might occur more frequently than is assumed, given that only 1 in 4 million individuals are born with HGPS [4].

The inner cells almost instantly divide to form the epiblast and hypoblast (also known as primitive endoderm in mice) [18,23]. The primitive endodermal (PE) cell nuclear shape analysis in murine blastocysts revealed a nuclear volume reduction and a uniform condensed ovoid shape, which coincided with elevated Lamin A/C levels in PE differentiation from murine ES cells in vitro [21]. The interruption of *LMNA* gene expression during PE specification in vitro impedes the endodermal differentiation [21], which indicates the activity of the mutated *LMNA* gene during the hypoblast specification in embryogenesis of HGPS might hamper the development of embryonic membranes due to progerin toxicity. During implantation, the hypoblast cells become part of the extraembryonic yolk sac [24], coinciding with the appearance of the amniotic folds [23]. Interactions between the emerging extraembryonic tissues and the epiblast cells initiate the formation of the primitive streak at the posterior end of the epiblast, marking the onset of gastrulation and the development of the three primary germ layers [23,25,26].

### 2.2. Lamin A Performance in Gastrulation

Gastrulation is the process of a primitive streak formation from the low Lamin A/C [20] pluripotent epiblast cells by mechanical forces released from cellular dynamics, which push bottle-shaped cells with apical constriction to ingress [27]. Low Lamin A pluripotent epiblast cells delaminate from the primitive streak, undergo epithelial-to-mesenchymal transition (EMT), and acquire the distinct mesoderm line specification [18,25,28,29].

Shortly before the start of gastrulation, the division rate of epiblast cells increases to gain critical cell mass in the region of primitive streak formation [30], resulting in increased cell density and cell–cell pressure. Consistently, some of these cells acquire bottle-shaped morphology and must be squeezed out through decayed underlying basal lamina [27]. Cell migration through narrow spaces should be accompanied by nuclear deformation, with Lamin A/C serving as the primary resisting component [31]. In relation to Lamin A turnover dynamics, it has been shown that increased Lamin A phosphorylation precedes lamina disassembly during mitosis. Similarly, elevated Lamin A phosphorylation has been revealed under relaxed conditions, thereby favoring Lamin A disassembly, which results in nuclear softening [32,33]. Thus, as actomyosin cytoskeleton relaxation in ingressing bottle-shaped cells [34] reduces tension on the nucleus, it is expected to respond with softening, accommodated by a reduction in Lamin A in the nuclear lamina. In the case of HGPS, the progerin-incorporated nucleus might not soften, as the progerin conformation alters its phosphorylation and, consequently, turnover, resulting in nuclear stiffening and rupture. Therefore, mesoderm formation might be negatively affected by developing embryos with the *LMNA* gene mutation.

### 2.3. Mesoderm-Derived Affected Tissues

Delaminated cells during gastrulation undergo subsequent EMT and migrate in the space between the visceral endoderm and the epiblast, acquiring distinct mesoderm line specifications [18,25,28,29]. The first mesoderm lineage to emerge is extraembryonic; the subsequent lineages ingress through more anterior regions at a later stage: lateral plate mesoderm, intermediate mesoderm, paraxial mesoderm, and axial mesoderm [18,25]. Lateral plate mesoderm (LPM) and paraxial mesoderm-derived cell types will be further considered in relation to HGPS disease.

HGPS manifestation extensively occurs among LPM and paraxial mesoderm derivatives. LPM domain, referred as second heart field (SHF) gives rise to vascular smooth muscle cell (VSMC) population at the base of the aorta, and other LPM domains contribute to the smooth muscles most of the other parts of the vascular system [35,36,37], while paraxial somitic mesoderm-derived smooth muscles are presented less in areas comprising the vasculature of vertebrae-associated vessels and dorsal aorta [38,39]. As cardiovascular diseases are referred to as the main cause of death in most HGPS patients at a mean age of 13, it is crucial to determine the exact structures responsible for such complications [4]. Although HGPS-affected children are usually born without cardiovascular deviations [4], the early onset of processes associated with vascular aging leads to increased risk of myocardial infarction and strokes at a young age [7]. Patients’ examination data show hypertension, aortic and mitral valve insufficiency or stenosis, compensatory left ventricular hypertrophy, and arterial occlusion [40,41] (connection with mesodermal development and progenitor mesodermal types). Autopsy and histological results are consistent with lifetime examinations and reveal severe atherosclerosis of the aorta and other arteries, expressed in the thickening of the intima and adventitia with Type 1 collagen accumulation, whereas the media are significantly depleted of VSMCs and replaced with fibrous tissue. There are also observed areas of calcification and abnormal extracellular matrix accumulation in the vessel and valve walls [42,43,44]. Moreover, the expression of human progerin in transgenic mouse models imitates HGPS symptoms [45,46] and causes the failure of specific aorta regions [47], which was reported in HGPS patients to be atherosclerosis [42] because of VSMC depletion [43]. Therefore, extensive atherosclerosis in HGPS patients results from depletion and loss of function of vascular smooth muscles, which are of LPM and paraxial mesoderm origin.

Apart from vascular disease, all HGPS patients suffer from subcutaneous fat loss [1,4,6,48], which causes insulin resistance and sclerodermatous skin changes [1,2,3,4,6,49]. Dermal fibroblasts (dFbs) and adipocytes, as the prominent skin residents, are also known to originate from LPM and the paraxial mesoderm. In addition, dFbs from HGPS patients demonstrate premature senescence by expressing pro-inflammatory factors in culture [50]. It is shown that senescing HGPS Fbs have a reduced ability to differentiate into adipocytes [49]. Progerin toxicity, in conjunction with pro-inflammatory factors in Fbs, may interfere with adipose tissue development. The dermomyotome of the somite divides into myotome and dermatome, with the latter giving rise to the dorsal dermis, while limb and ventral connective tissues, including dermis cell populations, are of LPM origin [37,38,51]. At the early stages, this condition is characterized by thickened corium, abnormal collagen accumulation, and loss of Fbs. As the disease progresses, the epidermis becomes thin, and the corium is replaced by fibrotic hyaline tissue [4]. Sclerotic skin is accompanied by discolored and pigmented stain appearance [2,3,6], extensive hair loss [3,5,48], and nail dystrophy [4]. Therefore, sclerodermatous skin changes and associated alterations originate primarily from dFbs dysfunction, which is mesoderm-derived.

Severe bone malformations are another pronounced complication in HGPS. Bone tissue of the body is known to be a mesoderm derivative, with the LPM giving rise to the skeleton of the limbs and the sclerotome of the somite producing the axial skeleton (vertebrae and ribs), the vasculature of the vertebrae, associated vessels, and the dorsal aorta [38]. However, the bones of the skull in HGPS patients show the most significant changes, while the bones of the axial and appendicular skeletons are less impacted. Bone-associated alterations typically include insufficient ossification of the neurocranium [2,4], as well as generalized osteopenia or osteoporosis [1,5,7,48]. Beaked nose and micrognathia are among the most common features in patients’ appearance [1,3,6,48], which could be a result of either underdevelopment of viscerocranium, especially jaw bones [7,48] or osteolysis [4]. Bone tissue resorption also occurs in distal phalanges, acromial parts of the clavicles, and the first ribs [2,4]. Jaw bone malformations seem to implicate tooth eruption and disposition [4,48]. Although its structure, in some cases, is unimpaired, hypodontia and enamel hypoplasia may be present [1,6,48]. In some patients, conductive hearing loss is observed at low frequencies [1,52,53]. This complication may occur due to a range of factors or their combination, which include osteolysis of certain parts of the temporal bone and middle ear bones, alterations in subcutaneous and other connective tissues, and tympanic membrane stiffening [2,52,53]. Thereby, the mesoderm-derived bone component in HGPS is not affected as extensively as in the case of vessel walls and skin dermis, although the chondrogenic and osteogenic specification potential of mesoderm cells seems to be restricted.

Clinical observations apparently confirmed HGPS disease to be associated with the mesoderm germ layer, although some of the mesoderm-derived tissues stay unaffected. For instance, the urogenital system, which originates from the LPM domain and intermediate mesoderm, shows no signs of the disease [2,37]. LPM and myotome-derived striated muscles seem primarily unimpaired, although experiments on the HGPS mouse model provide some evidence of loss of function, which, nevertheless, may develop due to vascular alterations and a general unhealthy condition [2,54]. A similar situation may be observed in the heart of HGPS patients, where structures and cardiomyocytes arise from LPM domains, the first and second heart fields (FHF and SHF) [37]. LPM-derived hematopoietic lineage is not affected as well, since there are no deviations in hematological parameters in the observed HGPS patients [1,55]. Additionally, all ectoderm- and endoderm-derived tissues exhibit the absence of HGPS manifestation, despite the *LMNA* gene being expressed in mammalian organs of endo- and ectodermal origin [56]. Neurological development in patients is unimpaired, along with the gastrointestinal tract and associated organs [2]. Despite the progerin expression, these mesodermal, ectodermal, and endodermal derivative tissues do not struggle with HGPS manifestation.

Thereby, the analysis of pathophysiology reveals that all clinical manifestations of HGPS are confined to a common developmental origin, the mesodermal germ layer, as there is a complete absence of evidence that any organ or tissue of ectodermal or endodermal origin is affected by the syndrome [2]. While the urogenital system, cardiomyocytes, blood, body musculature, and some other tissues of mesodermal origin preserve a clinically normal state, among the mesoderm derivatives, there are only three mesenchymal cell populations that are primarily compromised in HGPS: VSMCs, Fbs of the dermis layer, and osteogenic and chondrogenic cells. It is also important to emphasize that all of these cell groups, highlighted by clinical characterization, represent widespread populations that are not always exclusively of mesodermal origin. In different body parts, these populations are represented by cells that arise either only from the mesoderm, only from the neural crest, or from both simultaneously. Therefore, we hypothesize that HGPS does not affect all mesodermal lineages and derivatives equally but rather manifests only in tissues exhibiting developmental heterogeneity involving neural crest cells.

To investigate possible explanations for these similarities between mesoderm and neural crest mesenchymal cell populations affected by the HGPS, we now address the early embryogenesis of the neural crest progenitor cell population.

### 2.4. Neural Crest Development and HGPS-Affected Tissues

Following gastrulation, the next significant event in embryonic development is neurulation, resulting in the formation of the neural tube and the separation of the epidermis, the neural crest, and the craniofacial placodes. Although the division of the presumptive neural and non-neural ectoderm occurs shortly before gastrulation begins, the formation of the neural plate only starts after gastrulation is complete, in response to inductive signals from the extraembryonic tissues and underlying mesoderm [57]. As neurulation proceeds, the neural crest cells reside in the neural folds, and then undergo an EMT, acquire migratory abilities, and leave the dorsal neural tube region, where progenitor roof plate cells take the vacant place [57,58,59]. The newly formed neural tube consists of progenitor neural cells (PNCs) with typical apico-basal polarity in a pseudostratified epithelium layer with a central lumen [59,60]. Neural crest cell delamination occurs along the anteroposterior axis and proceeds in the posterior direction of neural tube closure, coinciding with an increase in mitotic activity, which results in cell-to-cell compression [30]. Surrounding cells of the dorsal region of the neural tube, where neural crest cells are about to delaminate, exhibit higher tissue stress, in contrast with previous and subsequent regions [61]. Neural crest cell delamination and EMT occur concurrently with basal lamina delamination, although some of the exiting cells initially display an atypical round morphology. This process enables neural crest cells to acquire mesenchymal characteristics and motility [61]. Neural crest cell migration is a strictly coordinated migration, whose path runs between the ectoderm layer and the underlying mesoderm, and thereby neural crest cells invade non-neural crest-derived tissues [62]. Investigations on *Xenopus laevis* embryos have shown neural crest cell migration to be dependent on the mechanical properties of the underlying mesoderm, as neural crest cell migration is activated in response to increasing mesoderm stiffening, which arises due to local increase in density through convergent extension of mesoderm cells in the area underneath the pre-migratory neural crest cells, with contribution of actomyosin cell cortex state [63,64]. We assume that such mechanical strain from the underlying mesoderm might also be present and sufficient for the onset of mammalian neural crest cell delamination and migration. Therefore, in the case of HGPS, the neural crest cell nucleus might not respond properly to mechanical cues, which are highly significant in neural crest cell differentiation or migration, so that tissues would be underrepresented with neural crest-derived cells, causing the HGPS phenotype in them.

## 3. Heterogeneous Origin of HGPS Manifestation

To sum up, as described in Section 2.3, the axial skeleton of the mammalian body originates from the somite mesoderm, while limb bones and connective tissues are derivatives of LPM [37,38]. However, neural crest derivatives are known to make the most significant contribution to the craniofacial skeleton in mammals [65].

VSMCs reside in the medial layer of the blood vessel, providing it with mechanical properties and bearing functions of response to physiological stimuli. In birds and mammals, it has been demonstrated that VSMCs of the media in the dorsal aorta originate from the sclerotome of the somite [35,36]. Other sources of VSMCs are various domains of the lateral plate mesoderm: the pre-epicardial, a derivative of cardiogenic mesoderm, forms the smooth muscle wall of the coronary vessels, while the mesothelium, or coelomic epithelium, of the visceral leaflet of the lateral lamina also forms the smooth muscle walls of the vascular flow [35,36,37]. Another source is the existence of two distinct populations of progenitor cells, found in the medial layer and in the aortic adventitia [36]. However, not all VSMCs are of mesodermal origin; the neural crest also makes a significant contribution to the population of these cells. Neural crest-derived VSMCs make up the medial muscular wall of the ascending branch of the aorta and the branching proximal portions of the subclavian and carotid arteries [35,36]. At the same time, VSMCs at the very base of the aorta are derived from the secondary cardiogenic field of the lateral plate, while the wall of its descending arch is composed exclusively of cells derived from somite mesoderm [35,39]. Whether the LPM and paraxial mesoderm have differential *LMNA* expression or mechanosensitivity is a question for further exploration. However, there is strong evidence that the *T* gene (*Brachyury*), which is the main mesodermal inductor, is controlled by Lamin A during specification of mesodermal cells [66].

The dermis is a skin layer underneath the epidermis, with Fbs as a main constituent of its stratified structure. There is a thin papillary layer underlying the basement membrane of the epidermis, the central part is the reticular dermis with hair follicles and sebaceous glands, and the deepest layer is the hypodermis, which is composed of white adipose tissue [67]. dFbs are a heterogeneous population in morphology and function, a cell population; therefore, distinct developmental linages can be distinguished: the upper subpopulation of papillary Fbs is associated with hair follicles, arrector pili muscle, dermal papilla, and dermal sheath Fbs; and the lower subpopulation, which gives rise to reticular Fbs and hypodermal adipocytes [68,69]. Histological evidence of the medial smooth muscle cell layer of HGPS patients reveals fibrosis and extensive cell loss, whereas Fbs of adventitia are primarily unaffected [42]. Dermal Fb populations mostly develop as mesoderm derivatives, except for the facial Fbs that arise from the cranial neural crest cells [51]. In the dorsal trunk of mammals, Fbs originate from a cell population that delaminates through EMT from the dermomyotome of the somitic mesoderm; dermal Fbs of the limbs and the ventral part of the body are derived from LPM [51]. The neural crest also gives rise to the melanocyte cell population, which contributes to skin and cell pigmentation. Melanocyte progenitor cells have been shown to reside in the adipose tissue of the hypodermis [69,70].

## 4. Epithelial-to-Mesenchymal Transition in Development and HGPS Manifestation

Epithelial-to-mesenchymal transition (EMT) is a dynamic process through which cells undergo a range of intermediate states between epithelial and mesenchymal morphologies. The process entails alterations in the expression profile, impacting a broad spectrum of proteins, including those associated with cell adhesion and polarity [71]. Cells are bound to lose epithelial E-cadherin and apico-basal polarity and acquire front–rear polarity, which is related to a range of cytoskeleton rearrangements, to achieve a motile cell state [71]. As previously discussed in Section 2.2, EMT-associated cytoskeletal rearrangements in delaminating mesodermal and neural crest progenitors generate mechanical forces, which are transmitted to the nucleus. Therefore, the nucleus has to be independently considered with its physical characteristics in terms of HGPS disease under continuous mechanical forces during development.

Since the cell nucleus is the organelle surrounded by the nuclear envelope, it organizes chromatin with an ensemble of nuclear envelope-anchoring and DNA-binding proteins. This organization enables the separation of nucleoplasm and facilitates dynamic processes of chromatin reorganization that respond to external and internal stimuli by activating or inhibiting genes. Despite being a complex and crowded environment for transcriptional regulation, the cell nucleus bears physical characteristics of stiffness to react as a mechanosensor to internal/external forces, such as cell polarity, induction-differentiation, EMT, migration, and basal membrane (substrate) hardness in embryogenesis. Therefore, the dynamic volume changes and/or nucleus deformation are part of the normal physiological response of the cell nucleus to the surrounding mechanical forces. To manage the dynamic nucleus deformation/reorganization and handle mechanical forces, the inner nuclear envelope is covered with a filamentous meshwork of type V intermediate filaments.

### Progerin Accumulation Alters Characteristics of the Nuclear Lamina Architecture

The filamentous meshwork of an inner nuclear envelope constitutes a nuclear lamina from assembled tetrameric filaments of 3.5 nm thickness of A- and B-type lamins, which co-present in densely packed and sparsely occupied regions of lamina meshwork [72]. The physical characteristics of nuclear lamina filament length and persistence indicate flexibility, and further packaging into the ~14 nm-thick lamina indicates dense meshwork [72]. Therefore, the main constructive block of the cell nucleus, the nuclear lamina, is the dynamic architectural structure facing the mechanical forces during nuclear deformation/reconstruction and cell division in embryonic development.

The dynamic and mechanical deformation/reconstruction of the nucleus in EMT, cell migration, and cell polarity processes, which dependently bring the nuclear viscoelasticity, another property of the cell nucleus, was demonstrated to be 3–4 times stiffer and nearly twice as viscous as the cytoplasm [73]. Consequently, the cell nucleus is the medium of nano-to-micro multiscale viscoelastic organization, which is driven by the lamina, the chromatin network, and the nucleoplasmic fluid [74], to respond to internal/external, functional, developmental, and structural stimuli and processes of the cell.

The progerin toxicity of suffering tissues and their cells is elucidated in the postnatal development of HGPS patients. It is shown that the physical properties of HGPS Fbs nucleus are affected by progerin; consequently, the Lamin A-associated chromatin architecture and the associated gene expression profile change.

**Figure 2:**
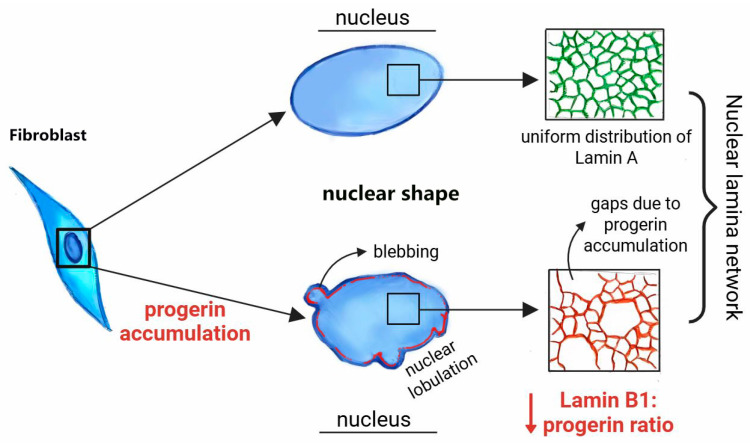
Progerin-associated nuclear malformations due to the alteration of the nuclear lamina in HGPS fibroblasts.

Despite the normal nuclear shape of 2-year-old HGPS Fbs at early in vitro culture, the developing malformation of HGPS Fb nucleus at late passages [75,76] confirmed the extensive lobulation, the loss of peripheral and internal heterochromatin, the abnormally clustered distribution of pores in the nuclear envelope, and the increase in the thickness and prominence of the lamina [75]. As a result, the nuclear stiffness of HGPS Fbs increases while the cytoskeletal organization of late-passage HGPS Fbs did not demonstrate defects and structural changes [76]. The elevated nuclear stiffness of HGPS cells occurs due to the immobility of progerin with its covalent binding to the inner nuclear membrane, so that the thickness of the lamina increases [77], which is accomplished by the simultaneous trap of abnormal progerin [78], normal Lamin A, Lamin C [79], and the slowly turning over Lamin B1 [80]. The demonstrated malformation of the HGPS Fb nucleus refers to the disruption of the nuclear architecture. Since a mutation in the *LMNA* gene disrupts the post-translational processing of Lamin A protein, it results in the accumulation of the mutant protein, progerin. Due to the carboxymethylated and farnesylated C-terminus, the progerin covalently incorporates into the nuclear lamina, which leads to a gradual increase in lobulation and nuclear malformation in the Fbs of patients in vitro (Figure 2).

Nevertheless, isolated Fbs are usually cultured on substrates whose physical properties differ from physiological, so that the mechanical characteristics of the substrate affect Fbs functioning. With an increase in substrate hardness, dermal Fbs in culture express higher levels of fibrosis-associated genes, relative to Fbs in native dermis [81]. Therefore, transcriptomic profile expression, chromatin accessibility, and further research conducted in vitro on HGPS-derived Fbs must be elucidated with the interpretation of the substrate hardness effect. In addition, increased hardness of a substrate in culture triggers actin cytoskeleton reorganization into stress fibers [82], which propagates mechanical stress to the nucleus through the LINC complexes [83]. Due to the elevated HGPS Fbs nuclear stiffness, the substrate-related hardness might have a cumulative impact on lobe formation and nuclear rupture. Induced senescence of normal pulmonary Fbs in culture demonstrated increased levels of senescent phenotype hallmarks on rigid substrate [83], so that DNA damage and cell senescence might be strengthened in already-aging HGPS Fbs in vitro.

The undergone gain and loss alterations in chromatin accessibility of the genomic regions in HGPS dFbs are associated with Lamin A. The differentially accessible chromatin regions of HGPS dFbs are significantly underrepresented with active chromatin marks H3K4me3, H3K27ac, and H3K36me3, while they are overrepresented with the repressive chromatin mark H3K9me3 [84]. Consequently, the differential chromatin accessibility points to the epigenetic alterations associated with DNA methylation and loss of heterochromatin-associated histone marks at the nuclear periphery of HGPS dFbs [85]. While overall median methylation levels demonstrated a minor increase in HGPS dFb, the nuclear lamina-associated domains exhibited a substantial and significant increase in methylation levels [85]. In addition, age-related partially methylated domains (PMDs) in HGPS dFb demonstrated elevated methylation levels [85]. However, CpG island methylation in the HGPS dFb was unchanged. In addition, the H3K9me3-associated histone modification at hypermethylated genome regions may be regulated by the de novo methylation of DNMT3B, as it is enriched in Lamin A-associated genomic regions [85]. The chromatin access alterations are accompanied by methylation deregulation, histone modification changes, and intranuclear relocalizations of genes, which drive differential gene expression in HGPS dFb.

The physical nuclear malformation of HGPS dFbs brings alterations in chromatin accessibility of genomic regions at the nuclear periphery and the enrichment of lamina-associated transcription factors, resulting in differential expression of genes responsible for “organismal” and “developmental” processes as well as “signaling” and “cell communication” [85].

Progerin accumulation in HGPS cells results in nuclear malformation due to the alteration of the nuclear lamina, causing complete loss of peripheral heterochromatin in these nuclei. The genome of compartment A, where progerin localizes, in the HGPS nucleus of Fbs, is enriched with differential chromatin accessibility, which is accompanied by epigenetic and gene expression alterations. However, revealed differential chromatin accessibility, altered DNA methylation, and differential expression in HGPS Fbs in vitro do not portray the processes in development and cannot be applied to explain HGPS manifestation in utero. Therefore, we recommend that researchers reconsider the strategy for investigating HGPS disease development.

To sum this up, the HGPS manifestation and tissue development point to the origin of two cell populations and their stochastic and heterogeneous coexistence. Symptoms of HGPS patients brought us to the mesodermal and neural crest cell populations in early development. Nevertheless, being derivatives of mesoderm and neural crest cells, not all tissues exhibit HGPS manifestation. However, the heterogeneous and stochastic combination of these two cell populations reveals the primarily affected cell populations in tissues: SMCs of the vascular medial layer, dermal layer cells, and osteogenic and chondrogenic cells of bone tissue. The EMT and migration are distinguishing properties of these two cell populations, so that mesodermal and neural crest cells are continuously subjected to mechanical stimuli from surrounding cells and ECM throughout development. The mechanical stimuli are accommodated and transferred through the cytoskeleton to the nucleus, which responds to mechanical forces through the resistance of the nuclear lamina meshwork. However, the viscoelastic property and the mechanosensor capability to respond to mechanical stress of the HGPS cell nucleus are disturbed. Therefore, the HGPS manifestation would have been expected in early developmental stages, which is not happening, and relatively healthy babies are born.

## 5. How and Why Are Infants with the *LMNA* Gene Mutation Born with the Absence of Any Severe Deviations?

Levels of expression of A-type and B-type lamina proteins differ across developmental stages and among differentiated adult tissues, with B-type lamins present from the earliest stages in undifferentiated embryonic cells, and A-type lamins are expressed in a differentiated tissue form [86]. Although *LMNA* expression is detected in vitro in differentiating mouse embryonic stem (ES) cells before the downregulation of pluripotency marker *Oct4* expression, its expression might be considered either as an early sign of differentiation in vitro or that A-type lamins’ presence may not be restricted exclusively to differentiated cells [87,88]. Moreover, there is evidence that A-type lamins are detected at the nuclear periphery in both trophoectoderm cells and pluripotent inner cell mass (ICM) of the mouse blastocyst, suggesting that A-type lamins are located in the nucleus of cells from the earliest stages of embryonic development in vivo along with B-types [88].

Differentiated cells exhibit cell-specific composition of nuclear lamina proteins; therefore, developmental and physiological processes require mechanisms for nuclear lamina protein regulation and exchange [86]. Lamins frequently undergo phosphorylation by specific sites, among them serine-404 in Lamin A, which is phosphorylated by AKT kinase and is known to promote prelamin A degradation [79]. Tissues affected by the HGPS mutation with age exhibit a significant increase in the levels of the mutant protein, progerin. However, a high amount of progerin is not established by elevated transcript levels of the mutated *LMNA* gene, suggesting that progerin accumulation occurs due to its inability to be exchanged and turned over [79]. Therefore, the reason for progerin accumulation in aging mice of the HGPS model is the reduced activity of the AKT kinase [78] and lower levels of serine-404 phosphorylation in progerin compared to prelamin A and normal Lamin A [79]. The PI3K/Akt signal transduction pathway, which promotes growth and cell survival, exhibits progression from the zygote to the blastocyst stage in mice [89]. We assume that increased AKT activity during preimplantation embryogenesis might enhance the turnover of type A lamins, ensuring the survival of the preimplantation embryo. Taken together, the slow turnover of progerin itself and its adverse effect on the turnover of other nuclear lamina proteins result in elevated nuclear stiffness, lobulation, and, consistently, nuclear rupture upon cyclic stretching of SMCs, which triggers senescence and cell death.

Thus, even though Lamin A is expressed during embryonic development and, in the case of HGPS, its mutant form, progerin, tends to accumulate in the cell nucleus, causing DNA damage, nuclear malformation, and syndrome manifestation, how and why are infants with *LMNA* gene mutation born with the absence of any severe deviations?

Investigations conducted on mouse models of HGPS and analysis of the temporal expression of A- and B-type lamins might shed light on the nature of this phenomenon. HGPS mutation causes an extensive loss of medial VSMCs in the aorta, whereas such loss is not observed in cardiomyocytes, where the *LMNA* gene is also expressed. This difference in cell survival might be due to lower levels of *LMNA* expression in the heart compared to the aorta walls [79]. However, even in low levels, long-lasting expression would have eventually led to the same consequences in the heart as in the aorta walls, due to perturbed progerin turnover. However, there is no clinical evidence that a similar loss of cardiomyocytes occurs in HGPS patients with aging. Apart from lower levels of *LMNA* expression, cardiomyocytes differ from VSMCs in their Lamin A:Lamin B1 ratio, which is lower in heart tissues compared to the higher ratio observed in aortal walls [90].

Additionally, aortal intima and adventitia cells exhibit similar levels of *LMNA* expression as medial cells; however, unlike VSMCs, intimal endothelial and adventitial cells show no signs of pathology. These layers also exhibit significantly higher Lamin B1 expression compared to the medial layer [90]. Significantly, the higher Lamin A expression was observed in other tissues that are coupled with HGPS pathology, bone, and skin [90]. In the case of dFbs, expression of LBR, which is the Lamin B receptor protein, is observed in mice until embryonic day 16 (E16). In contrast, Lamin A is continuously expressed from E12 and postnatally [91], indicating that the Lamin A:Lamin B ratio in Fbs increases after birth and with aging. In addition, the loss of Lamin B1 has been shown to be associated with cellular senescence [92].

In the mouse model of HGPS disease, SMCs were found to have an elevated quantity of human progerin, which correlates with the reduced expression of the *LMNB1* gene and the amount of Lamin B1 protein in the nuclear lamina of the aging HGPS mouse model [78]. As a result of progerin accumulation, the structure of Lamin B1 changes, and gaps with irregularly large openings appear in the progerin-integrated abnormal lamina meshwork [79] (Figure 2). Such an altered nuclear lamina demonstrates the lobulations that were aligned with a high progerin:Lamin B1 ratio in HGPS Fbs at late passages [77]. Nevertheless, in progerin-expressing and farnesylated prelamin A-expressing HGPS mouse model cells, Lamin B1 has similar levels, which means the accumulation of progerin does not trigger Lamin B1 downregulation. Furthermore, under mechanical stress, the abnormal nuclear lamina was unable to condense after successful swelling, demonstrating stiffness and collapse of the HGPS nucleus, while in the wild-type nucleus, the peripheral lamina smoothly distributed the mechanical force [80]. The decreased representation of Lamin B1 at lobulation sites and decreased *LMNB1* gene expression in HGPS cells trigger and increase the nuclear membrane rupture in the mouse SMC HGPS model, especially at bleb sites of nuclear membrane [79], so that balanced representation and ratio of Lamin B1 and progerin might increase the capability of nuclear elasticity and decrease the nuclear stiffness.

Several mouse models of HGPS were developed. Although each was based on molecular mechanisms of the disease, none of them fully reproduced the HGPS phenotype observed in humans. Thus, aortas from adult progerin-expressing mice exhibited an extensive loss of SMCs, which is absent in farnesyl-prelamin A-expressing mice of the same age, whose aortal walls are normal [79]. Levels of progerin and farnesyl-prelamin A were equal in aortas from young mice of both models; however, in older mice, levels of progerin were shown to be increased, while farnesyl-prelamin A levels were slightly decreased. At the same time, Lamin B1 levels in both models showed a significant decrease in older mice, resulting in a difference in the ratio of progerin, farnesyl-prelamin A, and Lamin B1 in mice of different ages. Thus, progerin:Lamin B1 and farnesyl-prelamin A:Lamin B1 ratios are similar in aortas from young mice, but in older mice, progerin:Lamin B1 ratios are significantly higher. However, cultures of SMCs with induced expression of either progerin or farnesyl-prelamin A both exhibit a similar percentage of nuclei with morphological abnormalities and equal rates of cell death when subjected to cyclic stretching [79]. These inconsistencies in the effects of farnesyl-prelamin A in culture and in the mice aortas may arise from differences in Lamin B1 levels, in the way that cultured SMCs might have initially contained low levels of Lamin B1, such as in older mice. Induced expression resulted in a high farnesyl-prelamin A:Lamin B1 ratio, which was not observed in old mice, due to the natural decline of farnesyl-prelamin A levels. Lowered level of Lamin B1 in the aorta might either occur under conditions of normal aging or be accelerated by the presence of progerin.

**Figure 3:**
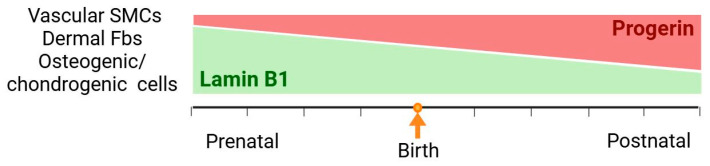
Progerin:Lamin B1 ratio in cells of affected tissues during prenatal and postnatal development may determine the delayed onset of HGPS after birth.

Therefore, a high ratio of progerin:Lamin B1 in cells of affected tissues may be responsible for the more severe manifestation of HGPS, and changes in this ratio during prenatal and postnatal development may determine the delayed onset of HGPS after birth (Figure 3). We also hypothesized that progerin toxicity might be managed and prevented, so that it could be rescued by the presence of Lamin B1.

## 6. Conclusions

The HGPS manifestation and tissue development indicate the origin of mesodermal and neural crest cell populations, as well as their stochastic and heterogeneous coexistence. The possible moment of syndrome onset after implantation is considered to be the EMT and gastrulation, when mesodermal and neural progenitors delaminate. The delamination of mesodermal and neural crest progenitors generates mechanical forces by cytoskeletal rearrangements, which are transmitted to the nucleus. However, the viscoelastic property and mechanosensor capability of the HGPS cell nucleus to respond to mechanical stress are disrupted due to the accumulation of nuclear lamina progerin protein, resulting in progerin toxicity and manifestation of the HGPS phenotype. Despite the possible intervention of TE specification and/or STB nuclear syncytialization, and the hypoblast specification by progerin toxicity in preimplantation embryo development, relatively healthy infants are born with HGPS. The cells in development undergo and face relatively more physical and mechanical stresses and cell divisions involving the nuclear lamina, especially Lamin A in differentiated cells, so that the accumulation of progerin and all type-A lamins will occur and demonstrate the insufficiency of the nuclear lamina. Accordingly, we have observed that the *LMNA* gene expression, in development, is spatially and temporally regulated in the developing tissues. In addition to the progerin:Lamin B1 high ratio in cells of tissues, which may be responsible for the more severe manifestation of HGPS, changes in this ratio during prenatal and postnatal development may determine the delayed onset of HGPS after birth. We also hypothesized that progerin toxicity might be managed and prevented, so that it could be rescued by the presence of Lamin B1.

One possible solution is to scrutinize the role of Lamin B1 in the coexistence of progerin during in utero development of an embryo and, consequently, the emerging capability of the cell to respond to mechanical forces on the nucleus. Therefore, the early developmental stages of the HGPS embryo can be imitated by synthetic embryology, which is a branch of embryology that deals with the generation of “stem-cell-based embryo models” (SCBEMs) from pluripotent stem cells [93].

The reason for writing this manuscript was the absence of a conceptual explanation of how the HGPS disease might manifest in utero. We consider pre- and postimplantational events in the context of *LMNA* gene activation and their contribution to cell fate specification and cell plate development, thereby addressing the hypothetical assumptions regarding the impact of its mutated protein on the in utero manifestation of HGPS disease. In the manuscript, we attempted to highlight each critical and decisive point of development at which the mutated *LMNA* gene might interrupt embryo growth. Firstly, upon continuous mechanical forces on the nucleus, cells dynamically respond by rearranging the nuclear lamina. The mechanical cues on cells alter the expression profile and behavior of cells and cell plates during development. The TE and hypoblast specifications are evident examples in which Lamin A expression perturbation brought the failure of their formation. The mechanical cues directly affect the nucleus and gene expression profile through the nuclear lamina, in which Lamin A is an active component involved in euchromatization and heterochromatization of peripheral chromatin. The empirical data obtained regarding the progerin effect on chromatin accessibility and the sequestration of transcription factors revealed that these changes occur due to morphological alterations in nuclear appearance, which are a consequence of alterations in the nuclear lamina in response to the cumulative impact of mechanical stress and progerin toxicity. In addition, this paper focuses on several aspects of developmental processes, including the mechanical impact on cell fate and cell plate formation, as well as the contribution of Lamin A in cell specification through the silencing and activation of genes regulating cell signaling, such as those involved in the specification of TE, hypoblast, and mesodermal cells. Therefore, we aimed to present a comprehensive and holistic view of development and the potential in utero manifestation of HGPS. Nonetheless, each successfully overcome embryo process and developmental stage will encounter a new stage barrier where the mutant *LMNA* gene must be activated. However, the question that needs to be addressed is how the developing embryo with the mutant *LMNA* gene is capable of overcoming the progerin handicap and toxicity, so that a conceived infant with HGPS appears normal. In addition, we have repeatedly emphasized that the syndrome is not diagnosed until the age of 2; that is, making HGPS unpredictable until its manifestation. The unpredictability of the progeria syndrome raises concerns for many young parents. Despite the rare frequency of HPGS patients, we have raised the question and concern of the frequent mutation in spermatozoa, which can be diagnosed early.

## Data Availability

No new data were created or analyzed in this study.
